# Repurposing Auranofin for Oncology and Beyond: A Brief Overview of Clinical Trials as Mono- and Combination Therapy

**DOI:** 10.3390/ph18111628

**Published:** 2025-10-28

**Authors:** Doralice Giorgini, Lorenzo Chiaverini, Monica Viviano, Raffaella Belvedere, Silvia Salerno, Emma Baglini, Federico Da Settimo, Tiziano Marzo, Sabrina Taliani, Elisabetta Barresi

**Affiliations:** 1 Department of Pharmacy, University of Pisa, Via Bonanno Pisano 6, 56126 Pisa, PI, Italy; dgiorgini@unisa.it (D.G.); lorenzo.chiaverini@phd.unipi.it (L.C.); silvia.salerno@unipi.it (S.S.); federico.dasettimo@unipi.it (F.D.S.); tiziano.marzo@unipi.it (T.M.); 2 Epigenetic Med Chem Lab, Department of Pharmacy, Via Giovanni Paolo II 132, 84084 Fisciano, SA, Italy; mviviano@unisa.it; 3 Department of Pharmacy, University of Salerno, Via Giovanni Paolo II 132, 84084 Fisciano, SA, Italy; rbelvedere@unisa.it; 4 Institute of Clinical Physiology, National Research Council of Italy, CNR Research Area, 56124 Pisa, PI, Italy; emma.baglini@cnr.it

**Keywords:** auranofin, clinical trials, drug repurposing, cancer

## Abstract

Auranofin (AF) is an oral gold(I) compound with a well-known pharmacological profile, currently used in the treatment of some severe forms of rheumatoid arthritis. Over the last twenty years, AF has also been repurposed as an antitumor, antiviral, and antibacterial drug. In this context, this review provides an updated overview of all clinical trials investigating AF for the treatment of various pathologies, either as monotherapy or in combination with other agents. We started summarizing the rationale behind repurposing AF in oncology, including its ability to inhibit thioredoxin reductase (TrxR) and disrupt redox homeostasis, leading to selective cytotoxicity in cancer cells. Clinical data from trials across a range of tumors are reviewed, highlighting safety profiles, dosing regimens, pharmacokinetics, and observed therapeutic outcomes. Then, we discussed the synergistic effects observed when AF is combined with chemotherapeutics, targeted therapies, or immune modulators. Then, an overview concerning the trials involving AF in non-oncological settings is also provided. Despite promising preclinical results, clinical translation remains in early stages, with most trials still in phase I or II. Nevertheless, emerging evidence supports continued exploration of AF-based therapies to address unmet medical needs.

## 1. Introduction

**Figure 1:**
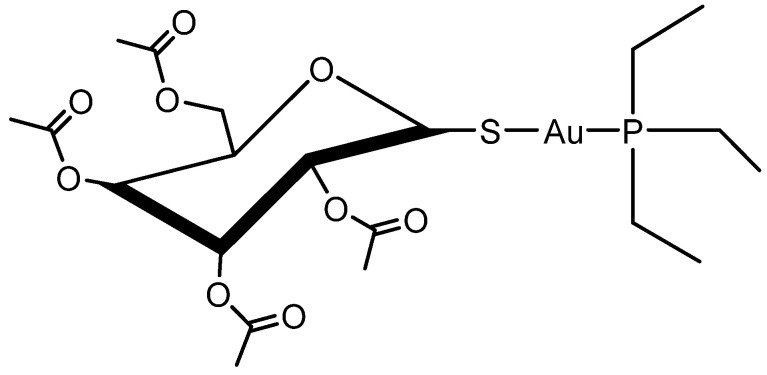
Chemical Structure of Auranofin.

Auranofin (AF, CAS 34031-32-8, molecular weight of 678.5 g/mol) is a linear gold(I)-based coordination complex that includes triethylphosphine and tetraacetylated thioglucose as ligands (Figure 1). Developed for the treatment of rheumatoid arthritis as an orally administered drug, AF (marketed as *Ridaura^®^*) received approval from the U.S. Food and Drug Administration (FDA) in 1985 as a first-line therapeutic agent [1]. However, its clinical application in rheumatology has diminished over time, mostly due to the advent of more effective synthetic and biological disease-modifying antirheumatic drugs (DMARDs), such as methotrexate, sulfasalazine, and adalimumab [2]. Recently, AF has gathered renewed attention due to its therapeutic potential in areas beyond rheumatology. Over the past decade, it has been widely repurposed and studied in multiple disease contexts, demonstrating potential efficacy in antibacterial, antiviral, antiparasitic, and particularly anticancer therapies [3,4,5,6].

**Figure 2:**
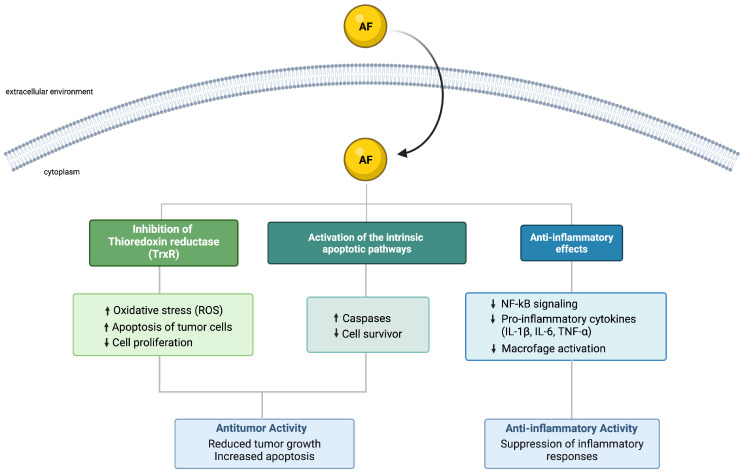
Mechanism of action of Auranofin.

AF is a prodrug, which activates in biological media, releasing its bioactive gold(I) species, mainly consisting of the [AuPEt_3_]^+^ cation. Importantly, the thioglucose moiety, though essential for solubility and transport, as well as for improved pharmacokinetics (PK) when orally administered, does not directly contribute to the therapeutic activity. In fact, it is the released [AuPEt_3_]^+^ cation that targets and inhibits thioredoxin reductase (TrxR), a selenoprotein deputed to the reduction in thioredoxin (Trx), Figure 2 [7]. There are two major isoforms of TrxR: TrxR1, located in the cytoplasm, and TrxR2, found in mitochondria. TrxRs are critical enzymes involved in maintaining cellular redox balance and supporting a range of physiological processes, such as DNA replication, regulation of gene expression, cell proliferation, and protection against oxidative stress-induced apoptosis [8]. The gold(I) ion released from AF strongly binds to the redox-active selenocysteine residue in the catalytic site of TrxRs, leading to irreversible enzyme inhibition. Mass spectrometry analyses indicated that each TrxR enzyme can coordinate up to four [AuPEt_3_]^+^ units, while biochemical studies confirm a profound disruption of the active site upon binding [9,10]. AF has also been shown to interact with hydroselenyl radicals (HSe)^−^, key intermediates in the biosynthesis of selenoproteins. These radicals are essential for the incorporation of selenium into selenocysteine, which is required for the proper function of a variety of selenium-dependent enzymes. AF can form a stable complex with HSe^−^, effectively sequestering these reactive selenium species. By trapping hydroselenyl radicals, it may reduce the intracellular selenium pool, thereby hindering the synthesis of selenoproteins. Since many organisms, including human cells and various pathogens, rely on selenoproteins for vital redox-regulating and metabolic functions, this interference can lead to impaired cellular function and reduced viability [11].

The TrxR/Trx system has gained significant attention in cancer biology due to its frequent upregulation in tumor tissues compared to healthy ones. This enhanced activity enables cancer cells to sustain a highly reduced intracellular environment, which facilitates survival under oxidative conditions, promotes tumor progression, and contributes to chemoresistance [12,13]. Notably, overexpression of the TrxR/Trx system has been associated with increased tumor aggressiveness and has emerged as a negative prognostic marker in several malignancies. These observations underscore the importance of redox-regulating enzymes not only in supporting malignant phenotypes but also in influencing clinical outcomes, highlighting the therapeutic potential of targeting redox homeostasis in oncology [13,14].

Based on this evidence, several clinical trials investigating AF have been initiated to evaluate its safety, efficacy, and therapeutic value in various cancer types (e.g., epithelial ovarian, primary peritoneal, and fallopian tube cancer [NCT01747798, NCT03456700], chronic lymphocytic leukemia (CLL) [NCT01419691], non-small cell lung cancer (NSCLC) [NCT01737502], etc.), either as monotherapy or in combination with other agents (Table 1). Beyond oncology, AF is currently being investigated in different clinical trials targeting non-oncological diseases, including parasitic infections and chronic viral diseases such as human immunodeficiency virus (HIV) (Table 1).

**Figure 3:**
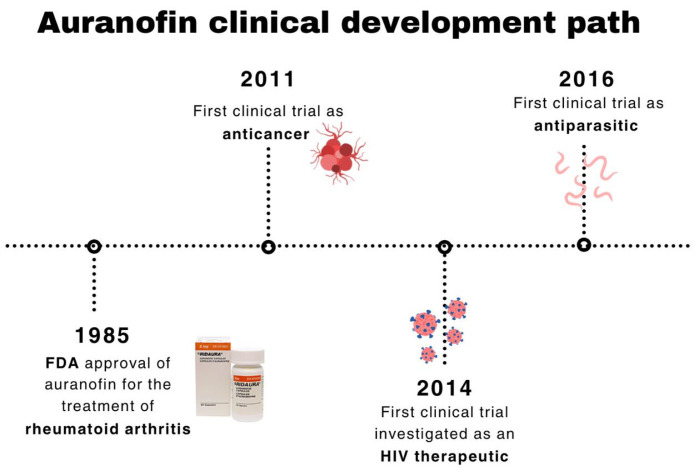
Timeline of AF’s clinical development path.

A timeline representation showing AF’s clinical development path is outlined in Figure 3.

Recently, several reviews dedicated to AF and its potential applications beyond rheumatoid arthritis have been published [1]. These reviews extensively summarize AF’s mechanisms of action, pharmacological profile, and preclinical evidence supporting its repositioning. The present report is focused specifically on the clinical outcomes of AF repurposing and explores, in particular, clinical evidence from both early-phase and ongoing trials involving various solid and hematologic tumors, examining the safety and tolerability of AF, optimal dosing strategies, PK, and preliminary therapeutic efficacy observed in diverse cancer types. Furthermore, clinical trials investigating AF in non-oncological diseases are discussed.

## 2. Auranofin-Based Monotherapy for Cancer Management

### 2.1. **NCT01747798**—Auranofin in Treating Patients with Recurrent Epithelial Ovarian, Primary Peritoneal, or Fallopian Tube Cancer https://clinicaltrials.gov/study/NCT01747798 (Accessed on 20 July 2025)

*Rationale*. Recurrent epithelial ovarian, primary peritoneal, and fallopian tube cancers remain a major clinical challenge due to the limited efficacy of standard treatments in the recurrent setting [15]. Ovarian cancer cells often exhibit a pro-oxidative status, meaning they have a higher level of Reactive Oxygen Species (ROS) than normal cells. This oxidative stress can contribute to the development and progression of the disease, including drug resistance and tumor metastasis [16]. AF is known to act as a pro-oxidant agent by inhibiting TrxR, with the ability to disrupt this antioxidant defense mechanism, leading to an accumulation of ROS and potentially inducing cancer cell death [17].

*Objectives*. The main objective of this phase 0 clinical trial was to evaluate the feasibility, tolerability, safety and preliminary efficacy of AF in patients with recurrent epithelial ovarian, primary peritoneal, or fallopian tube cancer, particularly focusing on those who were asymptomatic but exhibited elevated cancer antigen 125 (CA 125) levels, a biomarker associated with ovarian cancer recurrence [18]. More specifically, the trial aimed to assess the biochemical response rate, as measured by changes in CA 125 levels. By focusing on patients who showed rising CA 125 but no overt clinical symptoms, the study sought to determine if early intervention with AF could delay clinical progression.

*Treatment*. Patients received AF orally twice daily on days 1–28. No detailed information regarding the dosage of AF was provided. Courses were repeated every 28 days in the absence of disease progression or unacceptable toxicity. After completion of study treatment, patients were followed up every 6 months for two years.

*Eligibility*. The study enrolled 10 female patients aged 18 years or older who completed their initial treatment for epithelial ovarian, primary peritoneal, or fallopian tube cancer, which may have included surgery and/or chemotherapy, and who did not receive any further treatment for disease progression. A key inclusion criterion involved an elevated CA 125 tumor marker. Patients had to have an Eastern Cooperative Oncology Group (ECOG) performance status of 0–2, a standardized scale used to evaluate a cancer patient’s level of functioning. This criterion included patients ranging from fully active to moderately limited but still self-sufficient and ambulatory with adequate bone marrow, liver, and kidney function. Women of childbearing potential were required to have a negative pregnancy test prior to enrollment and must agree to use effective contraception during the study. In addition, all participants must be willing to provide informed written consent, take part in telephone interviews related to changes in CA 125 levels, and provide tissue samples for research purposes.

*Results and Conclusions*. The study successfully demonstrated the feasibility of enrolling and treating 10 asymptomatic patients with elevated CA 125 levels using AF. While specific efficacy outcomes were not detailed, the trial provided valuable insights into patient perceptions of CA 125 elevation and the potential role of AF in stabilizing or reducing CA 125 levels. These findings supported further investigation of AF as a maintenance therapy option for patients at high risk of recurrent disease.

### 2.2. **NCT01419691**—Phase I and II Study of AF in Chronic Lymphocytic Leukemia (CLL) https://clinicaltrials.gov/study/NCT01419691 (Accessed on 20 July 2025)

*Rationale*. CLL is the most common adult leukemia in Western countries, characterized by the accumulation of functionally incompetent B lymphocytes, which reside in the blood, bone marrow, lymph nodes, and spleen [19]. Although current therapies have improved patient outcomes, CLL remains incurable, and many patients eventually relapse or develop resistance to existing treatments. In this scenario, there is an ongoing need for new therapeutic strategies, particularly those that target the specific vulnerabilities of CLL cells [19].

AF showed preclinical efficacy against CLL by inducing apoptosis in CLL cells. This effect is achieved by triggering oxidative and endoplasmic reticulum stress, even in CLL cells with high-risk cytogenetic features like 11q or 17p deletions [20,21]. Given AF’s established safety profile in non-oncologic settings [22], repurposing it for use in CLL could represent a potentially cost-effective and innovative approach for treating this challenging disease.

*Objectives*. This clinical trial is structured in two phases—Phase I and Phase II—each with distinct but complementary goals aimed at evaluating the safety, tolerability, and preliminary efficacy of repurposing AF in patients with relapsed or refractory CLL, small lymphocytic lymphoma, and prolymphocytic leukemia. In Phase I, the primary objective was to determine the maximum tolerated dose of AF when given orally to patients by including careful assessment of the drug’s safety profile, potential toxicities, and dose-limiting side effects. In fact, establishing the appropriate dosage is essential to ensure patient safety and to define the optimal therapeutic window for subsequent evaluation. Following this, Phase II focused on assessing the clinical activity of AF at the identified dose by measuring the overall response rate, which included partial and complete responses based on established criteria for CLL. Additional objectives included evaluating progression-free survival and overall survival, as well as monitoring changes in biological markers that may reflect the drug’s mechanism of action—particularly its effects on oxidative stress, apoptosis, and redox regulation in leukemic cells.

*Treatment*. Patients received AF orally, 6 mg in the morning and 6 mg in the evening.

*Eligibility*. This study enrolled 15 patients who must have a histologically confirmed diagnosis of B-cell CLL, small lymphocytic lymphoma, prolymphocytic leukemia, or Richter’s transformation. Richter’s transformation is a serious complication of CLL, where the leukemia transforms into a more aggressive lymphoma, typically diffuse large B-cell lymphoma or, less commonly, Hodgkin lymphoma [23]. Only patients with relapsed or refractory disease who have received at least one prior line of treatment for CLL were eligible. Participants must be 18 years of age or older, have an Eastern Cooperative Oncology Group performance status of 0 or 1, and a life expectancy of at least 2 months. Furthermore, they must have adequate organ and bone marrow function.

*Results and Conclusions*. While specific results regarding response rates and adverse events were not detailed in the available sources, the trial’s completion suggested that AF was administered safely at the specified dosage. Further studies would be necessary to conclusively determine its efficacy in this patient population.

### 2.3. **NCT02063698**—Auranofin in Decreasing Pain in Patients with Paclitaxel-Induced Pain Syndrome https://clinicaltrials.gov/study/NCT02063698 (Accessed on 20 July 2025)

*Rationale*. Paclitaxel is a widely used chemotherapy drug, particularly in the treatment of breast, ovarian, and lung cancers [24]. Classified as a taxane, it works by interfering with the normal function of microtubules, which are essential for cell division, thereby preventing cancer cells from dividing and ultimately causing them to die [25]. One of its most common and distressing side effects is the development of paclitaxel-induced pain syndrome (PIPS), a form of neuropathic pain that typically arises within a few days after treatment and can severely impact a patient’s quality of life. This pain, often described as aching or burning, typically affects the hands, feet, legs, or back and may persist for several days to weeks after each cycle, limiting both patient comfort and treatment adherence [26]. Currently, there are no standard or effective treatments to prevent or alleviate PIPS, highlighting the urgent need to explore new therapeutic approaches to better manage this condition.

In this context, AF has been shown in preclinical studies to possess anti-inflammatory and neuroprotective properties, thanks to its ability to modulate oxidative stress and inhibit key pro-inflammatory pathways such as TrxR and the nuclear factor kappa-light-chain-enhancer of activated B cells (NF-κB), a protein complex that acts as a transcription factor regulating gene expression in response to various stimuli. It plays a crucial role in cellular processes like inflammation, immune responses, cell survival, and proliferation. NF-κB is involved in both normal physiological functions and pathological conditions, including atherosclerosis and cancer [27]. Given the suspected role of inflammation and oxidative damage in the development of PIPS, AF could represent a promising candidate for repurposing in this context.

*Objectives*. The primary objective of this clinical trial was to determine whether AF could effectively reduce the severity of PIPS in patients receiving chemotherapy. This study sought to evaluate whether one dose of AF could alleviate the intensity of pain symptoms, particularly in the days following chemotherapy, when pain tends to peak. To assess this, patients were asked to daily complete the Modified Brief Pain Inventory (mBPI) for 7 days [28]. The mBPI is a modification of the Brief Pain Inventory (BPI), a questionnaire used to evaluate the severity of a patient’s pain and the impact of this pain on the patient’s daily functioning, often used in clinical trials and research settings [28].

In addition to its primary goal, the study also included secondary objectives, such as evaluating the tolerability and safety profile of AF, monitoring for any side effects or adverse reactions. Furthermore, the trial might explore whether AF improves functional outcomes, such as physical activity levels, sleep quality, and the ability to carry out daily tasks during chemotherapy.

*Treatment*. Patients were randomly assigned to receive orally either AF or a placebo in a double-blind fashion. No specific details about the dose of AF have been published in the registry sources. Participants in the treatment arm received AF orally, starting within 24 h after paclitaxel administration. The drug was taken once daily for 7 days, which is the period when PIPS typically peaks. The control group followed the same schedule but received a placebo tablet instead of the active drug. The short treatment window was designed to specifically target the acute pain flare associated with each paclitaxel cycle, without interfering with the ongoing chemotherapy regimen. Severity and duration of pain between patients who received AF and those who received a placebo were compared to identify a statistically and clinically meaningful reduction in pain.

*Eligibility*. This trial enrolled 30 patients undergoing chemotherapy with paclitaxel for cancer. To be eligible for this study, participants must meet specific laboratory and clinical criteria. Women of childbearing potential must have a negative urine or serum pregnancy test performed within 7 days prior to registration, and all participants must be able to complete study questionnaires independently or with assistance. Importantly, participants must have previously experienced pain associated with paclitaxel administration, either during current or past treatment. Formal documentation of this prior pain episode was not required. Additionally, patients must be scheduled to receive paclitaxel at a dose of at least 70 mg/m^2^ within 14 days of randomization.

*Results and Conclusions*. In the phase II trial NCT02063698, eight distinct outcome measures were posted to evaluate the effect of a single dose of oral AF on PIPS. Although 30 patients were enrolled and randomly assigned to receive either a single oral dose of AF or a placebo on the day after paclitaxel administration, the study did not demonstrate a statistically significant reduction in overall pain scores. Although these differences were not large enough to meet conventional significance thresholds, the observed trend suggests a potential delayed benefit of AF over time.

## 3. Combinatory Regimens for Cancer Management Involving Auranofin

### 3.1. **NCT01737502**—Sirolimus and Auranofin in Treating Patients with Advanced or Recurrent Non-Small Cell Lung Cancer or Small Cell Lung Cancer https://clinicaltrials.gov/study/NCT01737502 (Accessed on 20 July 2025)

*Rationale*. Sirolimus, also known as rapamycin, is a macrolide compound originally isolated from *Streptomyces hygroscopicus*. It acts as an immunosuppressive agent by binding to FK-binding protein 12 (FKBP12) and subsequently inhibiting the mammalian target of rapamycin (mTOR) pathway, a central regulator of cell growth, proliferation, and metabolism. Owing to this mechanism, sirolimus has been widely used in clinical practice to prevent kidney transplant rejection and, when incorporated into drug-eluting stents, to reduce the risk of restenosis. Beyond its approved applications, sirolimus has been extensively investigated for its potential anticancer activity, as mTOR dysregulation is a hallmark in many tumor types, including lung cancer. Despite its broad therapeutic potential, sirolimus is associated with side effects such as hyperlipidemia, thrombocytopenia, delayed wound healing, and increased susceptibility to infections, which limit its long-term tolerability [29]. The interplay between AF and sirolimus is particularly relevant in cancer, as AF may enhance the effects of sirolimus. The oxidative stress triggered by AF may sensitize tumor cells to mTOR inhibition, enhancing the antiproliferative effects of sirolimus [30]. This synergistic effect is particularly relevant in chemoresistant tumors, where alternative survival pathways and redox adaptations contribute to treatment failure, and represents a promising strategy to overcome resistance and improve outcomes in patients with advanced or recurrent NSCLC and SCLC.

*Objectives*. This Phase I/II study was designed to explore the therapeutic potential of combining AF and sirolimus in patients with advanced or recurrent lung cancer.

The primary objective of Phase I of the trial was to determine the maximum tolerated dose of the combination therapy, in order to establish a safe and effective dosage regimen that can be carried out into further evaluation. In Phase II, the focus shifted to assessing the progression-free survival at 4 months of treatment, providing an early measure of the treatment’s clinical efficacy in this heavily pretreated population. The study also outlined several secondary objectives. These included evaluating overall survival of participants compared to controls, assessing the safety profile and adverse events of the regimen, and determining the overall response rate, as well as duration of response, in patients with measurable disease, based on Response Evaluation Criteria in Solid Tumors, a standardized system that assesses treatment efficacy through changes in tumor size on imaging studies. In addition, a key correlative research objective was to investigate the association between molecular biomarkers and clinical outcomes such as progression-free survival, overall survival, treatment response, and toxicity. This component aims to identify potential predictive markers that could guide future patient selection and personalized therapeutic approaches.

*Treatment*. The treatment protocol involved oral administration of AF every day from day 1 to day 28 of the treatment cycle. Sirolimus was also given orally from day 1 to day 28 but only starting from day 8 of the first course. No detailed information regarding the dosage of AF and sirolimus was provided. Courses were repeated every 28 days in the absence of disease progression or unacceptable toxicity. Following completion of therapy, patients entered a long-term follow-up phase with assessments scheduled every 3 to 6 months for up to five years. This extended follow-up period allowed for comprehensive monitoring of survival outcomes and late-onset adverse effects.

*Eligibility*. The study enrolled 29 patients. Eligible participants for this trial were required to have histologically or cytologically confirmed lung cancer, specifically squamous cell carcinoma, Ras-mutated adenocarcinoma, or SCLC. All patients must have previously received at least one line of platinum-based chemotherapy and must lack further standard treatment options. Patients were allowed to have had prior radiation therapy, provided they had sufficiently recovered from its toxic effects (excluding alopecia) before entering the study. Patients had to have an Eastern Cooperative Oncology Group performance status of 0–2 and a life expectancy of at least 12 weeks. Women of childbearing potential were required to have a negative serum pregnancy test within 7 days prior to enrollment. Additional criteria included providing informed consent, a commitment to return and enroll in the Mayo Clinic institution for follow-up, and a willingness to provide tumor tissue for related biomarker research.

*Results and Conclusions*. Although the clinical trial NCT01737502 has been completed, as of the latest update on 25 March 2024, no results have been posted.

### 3.2. **NCT03456700**—Auranofin and Sirolimus in Treating Participants with Ovarian Cancer https://clinicaltrials.gov/study/NCT03456700 (Accessed on 20 July 2025)

*Rationale.* As previously mentioned, AF induces oxidative stress and apoptosis in cancer cells by disrupting redox homeostasis, while sirolimus inhibits mTOR signaling. Preclinical data suggest that the simultaneous disruption of redox balance and growth signaling may exert synergistic antitumor effects. Importantly, this trial also enrolled patients based on the overexpression of protein kinase C-iota (PKC-ι), an oncogenic kinase implicated in tumor progression and poor clinical outcomes across multiple cancer types. PKC-ι has been shown to regulate both redox-sensitive and mTOR-related pathways, providing a biologically relevant target for dual inhibition using AF and sirolimus [31,32].

*Objectives*. The primary objective was to estimate the overall response rate across the entire study population. Secondary objectives included assessing the overall response rate specifically in the subgroup of patients whose tumors overexpress PKC-ι, as well as estimating progression-free survival, overall survival, and documenting adverse events associated with the treatment. In addition, the study included correlative analyses to explore whether PKC-ι-related biomarkers in tumor tissue are associated with treatment outcomes.

*Treatment*. The treatment regimen consisted of oral administration of AF and sirolimus once daily in 28-day cycles, which were continued until disease progression or unacceptable toxicity. After treatment completion, participants were monitored every 6 months for up to 3 years to assess long-term outcomes. No detailed information regarding the dosage of AF and sirolimus was provided.

*Eligibility*. This study enrolled 22 patients with incurable serous ovarian, fallopian tube, or primary peritoneal cancer, who had measurable disease and an Eastern Cooperative Oncology Group performance status of 0 or 1, indicating they were fully active or only mildly symptomatic. Patients were required to have adequate hematologic, hepatic, and renal function. A life expectancy of at least 12 weeks was also required. Importantly, participants needed to be willing to provide archival tumor tissue for biomarker analysis.

*Results and Conclusions*. Of the 22 patients enrolled, 21 completed the study, with one participant discontinuing early. The median overall survival was 4.4 months (95% Confidence Interval 2.6–12.5 months), and the progression-free survival was 2.1 months, suggesting limited efficacy of the combination therapy in this patient population. Regarding safety, 61.9% of participants experienced at least one Grade 3 or higher adverse event. Common serious adverse events included small intestinal obstruction (14.3%), vomiting (9.5%), and sepsis (9.5%), while non-serious ones were fatigue (90.5%), diarrhea (81%), and anemia (76.2%). In summary, while the combination of AF and sirolimus was generally tolerable, the modest clinical benefits observed did not support its use as a standard treatment for recurrent serous ovarian cancer.

### 3.3. **NCT02770378**—A Proof-of-Concept Clinical Trial Assessing the Safety of the Coordinated Undermining of Survival Paths by 9 Repurposed Drugs Combined with Metronomic Temozolomide (CUSP9v3 Treatment Protocol) for Recurrent Glioblastoma https://clinicaltrials.gov/study/NCT02770378 (Accessed on 20 July 2025)

*Rationale*. Recurrent glioblastoma multiforme poses a major clinical challenge due to its aggressive nature, poor prognosis, and limited response to conventional therapies [33,34]. This Clinical Trial investigated the CUSP9v3 protocol (Coordinated Undermining of Survival Paths, version 3), an innovative treatment approach that combines low-dose temozolomide with nine repurposed non-cancer drugs, including AF. Temozolomide is a small heterocyclic compound belonging to the imidazotetrazine family. Chemically, it is a prodrug that undergoes spontaneous hydrolysis at physiological pH, generating its active metabolite, which is responsible for the alkylating activity. Clinically, temozolomide has become the standard of care for glioblastoma due to its ability to induce DNA damage [35]. However, its efficacy is often limited by intrinsic and acquired resistance mechanisms, including DNA repair and metabolic adaptation. The combination of AF and temozolomide in the CUSP9v3 protocol is based on a strategy to overcome glioblastoma’s resistance to conventional therapies by simultaneously targeting complementary vulnerabilities within tumor cells. The inclusion of AF among the nine repurposed drugs used was based on the observation that glioblastoma cells possess strong antioxidant mechanisms to survive in hypoxic and metabolically stressful environments, while also displaying a notable sensitivity to disruptions in redox balance [35]. Thus, AF has been included in this trial due to its ability to disrupt redox homeostasis and increase oxidative stress in tumor cells, leading to apoptosis and reduced proliferation.

*Objectives*. The primary objective of this phase I/II clinical trial was to determine whether this multi-drug approach can be administered safely over time, with particular focus on identifying dose-limiting toxicities and establishing an optimal regimen that can be tolerated by most patients. Safety was monitored by tracking the incidence and severity of treatment-emergent adverse events, which were evaluated using the Common Terminology Criteria for Adverse Events, version 4.03 (CTCAE v4.03), a standardized system developed by the U.S. National Cancer Institute to classify and grade the intensity of adverse events. In addition to safety, the trial includes several important secondary objectives aimed at exploring the preliminary efficacy of the combination therapy, including measuring overall survival and progression-free survival, as well as evaluating objective tumor responses.

*Treatment*. In this investigational protocol, patients diagnosed with glioblastoma received temozolomide in combination with a multi-drug regimen composed of nine repurposed agents, each with a distinct pharmacologic profile and potential anticancer activity. The treatment begins with an induction phase lasting 35 days, characterized by a gradual and sequential introduction and up-titration of each individual drug. This phase is carefully structured to monitor tolerability, and adjustments are permitted based on individual patient responses and toxicity profiles.

The induction cycle initiates with temozolomide, administered at a low dose of 20 mg/m^2^ body surface area twice daily, and continues at this dosage throughout the entire treatment course. Aprepitant, a small molecule that acts as a neurokinin-1 (NK1) receptor antagonist, is introduced on day 1 at 80 mg once daily and maintained continuously. It belongs to the morpholine chemical class and is traditionally used as an antiemetic [36]. Despite its efficacy, aprepitant’s clinical use is limited by drug–drug interactions mediated by CYP3A4 metabolism and the restricted therapeutic indications. Subsequently, over the course of the induction cycle, additional agents are added in a staggered way. Minocycline, a tetracycline antibiotic with anti-inflammatory and neuroprotective properties [37], is initiated at 50 mg twice daily and escalated to 100 mg twice daily. It is characterized by a lipophilic chemical structure that allows for good tissue penetration and central nervous system access. Its clinical use is limited by resistance development, gastrointestinal side effects, and, in rare cases, drug-induced autoimmune reactions. Disulfiram, a thiuram-based small molecule that acts as an aldehyde dehydrogenase inhibitor with potential anti-tumor activity [38], follows a similar titration from 250 mg once daily to twice daily dosing. It was primarily developed for the treatment of chronic alcoholism, producing an aversive reaction upon alcohol intake. Although effective in promoting abstinence, disulfiram’s clinical use is limited by variable patient adherence, risk of hepatotoxicity, neuropathy, and severe reactions if alcohol is consumed during treatment, necessitating careful monitoring. Celecoxib, a diaryl-substituted pyrazole compound that acts as a selective cyclooxygenase-2 (COX-2) inhibitor known for its anti-inflammatory and anti-angiogenic effects [39], is introduced and escalated to 400 mg twice daily. Its clinical utility is limited by concerns over cardiovascular risks, potential gastrointestinal side effects, and drug–drug interactions, which require careful patient selection and monitoring during long-term use. Sertraline, a synthetic naphthylamine derivative that belongs to the class of selective serotonin reuptake inhibitors with reported pro-apoptotic effects in glioma cells [40], is increased from 50 mg to 100 mg twice daily. It was primarily developed as an antidepressant and is widely prescribed for major depressive disorder, anxiety disorders, and obsessive–compulsive disorder. Despite its broad efficacy, sertraline’s use can be limited by dose-dependent side effects such as gastrointestinal disturbances, sexual dysfunction, and insomnia, as well as the risk of withdrawal symptoms upon discontinuation and variable patient response due to genetic and metabolic differences. Captopril, a sulfhydryl-containing dipeptide analog that acts as a potent Angiotensin-Converting Enzyme (ACE) inhibitor that may impact tumor vasculature and immune modulation [41], is titrated to 50 mg twice daily. Its main limitation concerns the relatively short half-life, which necessitates more frequent dosing than newer ACE inhibitors. Itraconazole, a triazole antifungal agent with anti-hedgehog pathway and anti-angiogenic properties [42], is increased to 200 mg twice daily. Its clinical use, however, is limited by poor and variable oral bioavailability, significant drug–drug interactions due to CYP3A4 inhibition, and potential hepatotoxicity, which require careful monitoring and restrict its use in certain patient populations. Ritonavir, a peptidomimetic molecule belonging to the class of HIV protease inhibitors with the potential to inhibit P-glycoprotein and affect tumor drug resistance [43], is escalated to 400 mg twice daily. Its clinical application is limited by gastrointestinal and metabolic side effects, such as nausea, diarrhea, dyslipidemia, and insulin resistance, as well as by complex drug–drug interactions arising from CYP3A4 inhibition. Lastly, AF is introduced and increased to 3 mg twice daily. Following the induction period, patients proceeded to up to 12 treatment cycles, each lasting 28 days, during which the full combination was maintained at the target doses established during induction. The protocol allowed for individualized modification during the early treatment phases to optimize balance between treatment intensity and patient safety.

*Eligibility*. The eligibility criteria were designed to enroll 10 patients with histologically confirmed grade IV glioblastoma, including those with a prior diagnosis of lower-grade glioma who subsequently experienced histological transformation. Eligible participants had to demonstrate radiologically confirmed progression of disease, as defined by Response Assessment in Neuro-Oncology (RANO) criteria, standardized guidelines used to evaluate how brain tumors respond to treatment, following prior standard treatment with radiotherapy and temozolomide. To maintain consistency in disease staging, only patients who had experienced no more than three episodes of tumor progression were allowed to enroll. To mitigate the potential impact of recent therapies on trial outcomes, patients were required to have completed chemotherapy or surgical resection at least 4 weeks prior to enrollment and radiotherapy at least 12 weeks beforehand. Additional criteria included being over 18 years of age, having a Karnofsky Performance Status (KPS) of 70% or higher, a clinical scale used to measure a cancer patient’s functional ability and overall well-being, and being on a stable corticosteroid dose for at least one week prior to participation. Key hematologic and biochemical values also need to fall within specific ranges to ensure safety and treatment tolerability. Furthermore, patients had to be appropriately vaccinated (e.g., Pneumovax and varicella) and agree to contraceptive measures if of reproductive potential. All participants provided written informed consent and demonstrated an ability to comply with the study protocol.

*Results and Conclusions*. Of the 10 patients enrolled in the study, 9 were evaluable for the primary endpoint, which was safety. All these 9 patients successfully met the predefined safety criteria. Among the agents used in the combination regimen, ritonavir, temozolomide, captopril, and itraconazole commonly required dose adjustments or temporary discontinuation, primarily due to toxicity. The most frequently reported adverse events included nausea, headache, fatigue, diarrhea, and ataxia, consistent with known side effects of the included agents. Importantly, the progression-free survival rate at 12 months was 50%, which is notable in the context of recurrent glioblastoma. Based on these findings, the CUSP9v3 regimen appeared to be tolerable and feasible under close clinical supervision. These encouraging results have led to the planning of a randomized phase II trial to formally evaluate the regimen’s efficacy in a larger patient population [44].

## 4. Auranofin in Non-Oncological Diseases

While much of the current research has focused on its anticancer activity, AF is also being actively investigated in non-oncological settings. Three clinical trials, namely NCT02089048, NCT02736968, and NCT02961829, have explored its use in treating protozoal infections such as giardiasis and amebiasis, as well as chronic viral conditions like HIV, where its immunomodulatory and latency-reversing effects may offer therapeutic benefits [6,45].

### 4.1. **NCT02089048**—Auranofin Pharmacokinetic (PK) Following Oral Dose Administration https://clinicaltrials.gov/study/NCT02089048 (Accessed on 20 July 2025)

*Rationale*. Although AF is still in use for rheumatoid arthritis, most of the available pharmacokinetic data dated back to the 1980s and were based on long-term administration in chronically ill patients [6]. Moreover, older analytical methods lacked the sensitivity to accurately characterize gold distribution and elimination after short-course dosing [6].

Considering AF’s established mechanism of action and therapeutic potential, it became important to evaluate gold levels in healthy individuals following short-term administration, which better represents its intended use for parasitic infections more accurately than long-term dosing typically applied in autoimmune disorders. Building on strong preclinical findings and increasing interest in AF as a repurposed antiparasitic agent, including its orphan drug designation for the treatment of amebiasis, clinical investigation has become essential to guide its development for infectious indications [46].

*Objectives*. The primary objective of this Phase I clinical trial was to assess the PK of gold following a short-term dosing regimen in healthy adult subjects. This included determining key pharmacokinetic parameters, such as peak plasma concentration (C_max_), elimination half-life (t_1_/_2_), and fecal excretion, during the treatment period and through subsequent follow-up. The secondary objective was to evaluate the safety and tolerability of short-course oral AF in healthy volunteers. This involved monitoring adverse events, clinical laboratory values, and vital signs throughout and after the dosing period. The trial also aimed to quantify fecal and plasma gold concentrations to estimate drug distribution and elimination. These data are essential for establishing dosing strategies in future trials targeting gastrointestinal or systemic parasitic infections.

*Treatment*. Participants were enrolled at a single study site for this Phase I trial. Each subject received 6 mg of AF orally once daily for 7 consecutive days, which corresponds to the standard dosing approved for rheumatoid arthritis. The total duration of individual subject participation extended up to 23 weeks, which included a 7-day dosing period followed by long-term safety follow-up visits to monitor for delayed adverse effects and track the elimination of gold from the body. This extended monitoring was particularly important given gold’s known pharmacological persistence and tissue accumulation profile.

*Eligibility*. To ensure safety and uniformity in pharmacokinetic assessment, the trial enrolled 15 healthy adult volunteers who met clearly defined inclusion criteria. Participants were required to be male or female of non-childbearing potential, aged between 18 and 45 years, and in overall good health as determined by a comprehensive medical history, physical examination, and standard laboratory evaluations. Women were considered of non-childbearing potential if they were postmenopausal for at least two years with elevated FSH levels or had undergone a surgical sterilization procedure. This restriction helped eliminate risks related to potential teratogenicity. Participants needed to have a body mass index (BMI) between 18 and 30 kg/m^2^, and a body weight between 50 and 122 kg. In addition, male participants were required to agree to use appropriate contraception for the duration of the study to minimize reproductive risks associated with the investigational drug. All participants had to demonstrate a clear ability and willingness to comply with study procedures, including scheduled clinic visits, drug administration, laboratory testing, and safety assessments over the full duration of the study. These strict eligibility criteria ensured reliable pharmacokinetic data collection while maintaining subject safety in this first-in-population trial for antiparasitic use.

*Results and Conclusion*s. This Phase I clinical trial demonstrated that AF is well tolerated in healthy adult volunteers and provides a favorable PK profile for repurposing as an antiparasitic agent when administered during a limited treatment period. No serious adverse events were reported, and all treatment-emergent adverse effects were mild and self-limiting. PK analysis revealed a plasma C_max_ of 0.312 µg/mL on day 7 and a t_1_/_2_ of approximately 35 days, indicating slow systemic clearance. Importantly, high fecal concentrations of gold were achieved (up to 13 μM, or ≥25 times the IC_50_ for *E. histolytica*), suggesting that oral AF delivers therapeutically relevant levels to the intestinal lumen, the primary site of infection in amebiasis and giardiasis. These results support the feasibility and safety of short-course AF treatment in humans and provide essential pharmacological data to guide dose selection for future clinical trials targeting intestinal protozoan infections [6].

### 4.2. **NCT02736968**—Auranofin for Giardia Protozoa and Entamoeba histolytica http://clinicaltrials.gov/study/NCT02736968 (Accessed on 20 July 2025)

*Rationale*. The continued reliance on metronidazole as first-line therapy for anaerobic protozoan infections, including *Giardia intestinalis* and *Entamoeba histolytica*, has been challenged by emerging resistance, suboptimal efficacy against cystic forms, and the need for prolonged treatment regimens [47]. These limitations, combined with documented cases of clinical failure and laboratory-induced resistance [48], highlight an urgent need for alternative antiparasitic agents.

In this context, AF emerged as a promising candidate. It demonstrated potent in vitro activity against both *G. intestinalis* and *E. histolytica*, including metronidazole-resistant strains, with IC_50_ values significantly lower than those of metronidazole. Additionally, in vivo efficacy has been established in rodent models of amoebic colitis and liver abscess [49] as well as in Giardia infection models [50]. As already mentioned, AF targets TrxR, which is essential for protozoan survival and its repurposing as a broad-spectrum antiparasitic agent is further supported by efficacy data in other parasitic diseases [51,52].

*Objectives*. This Phase IIa clinical trial was designed to assess the efficacy and safety of AF in adults affected by two major protozoan infections: giardiasis and amebiasis. The primary objectives of the study were two. First, for patients with *G. intestinalis*, the trial aimed to determine the proportion of individuals who achieved clinical resolution of diarrhea (defined as fewer than three loose stools within 24 h) by day 5 of treatment. Similarly, for those with *E. histolytica*, the primary outcome was clinical resolution by day 7. These assessments were limited to individuals with confirmed infection via rapid enzyme immunoassay and antigen detection assays at enrollment. The secondary objectives were more extensive and included both parasitological and clinical endpoints. For both infections, the study aimed to evaluate (i) parasitological clearance, defined as the absence of trophozoites on microscopic examination or antigen detection at various time points (days 3, 5, 7, 14, and 28); (ii) reduction in trophozoite and cyst load in stool as measured by quantitative PCR (qPCR); (iii) sustained cure and relapse or reinfection, using molecular genotyping to distinguish between persistent or newly acquired strains; and (iv) the time to resolution of diarrhea as an additional measure of clinical response. These secondary outcomes allowed for a deeper understanding of the dynamics of infection clearance and treatment durability over a 28-day follow-up period. By combining traditional microscopy, molecular tools (qPCR, genotyping), and antigen assays, the trial was well-equipped to provide robust data on the effectiveness of a short course AF regimen.

Ultimately, this trial aimed to position AF as a novel, short-duration, oral therapeutic for protozoan infections, potentially overcoming the limitations of current standard-of-care treatments and offering a repurposed solution with a good safety profile.

*Treatment*. This Phase IIa trial used a randomized, single-blind, placebo-controlled design in adult patients diagnosed with either giardiasis or amebiasis. Participants were randomly assigned to receive 6 mg of AF orally once daily or a visually identical placebo. The treatment course was 5 days for those with giardiasis and 7 days for those with amebiasis. The total study participation for each subject spanned approximately 30 days, including a pre-enrollment screening period of up to 4 days and subsequent in-person follow-up visits.

*Eligibility*. A total of 136 participants (68 with giardiasis and 68 with amebiasis) were enrolled and randomized to receive either AF or a placebo. Participants eligible for inclusion in this trial were adults aged 18 to 65 years, including males and non-pregnant females. All subjects were required to have a confirmed diagnosis of either *G. intestinalis* or *E. histolytica* infection, verified through stool antigen detection and confirmatory laboratory testing at enrollment. In cases of dual infection, enrollment was directed to the *E. histolytica* arm unless that cohort was already full.

Participants were required to have recent diarrheal symptoms (≥3 loose stools in the past 24 h) while remaining clinically stable. Stable chronic conditions were permitted if unchanged in medication type, dosage, or frequency over the past 3 months and clinically well-managed in the last 6 months. Routine medications, including topical, inhaled, and contraceptive therapies, were allowed if not deemed to pose additional safety risk. Subjects had to present with normal vital signs and laboratory parameters within protocol-defined ranges, including assessments of renal and liver function, hematology, and urinalysis. Women of reproductive potential needed a negative pregnancy test within 72 h prior to study medication initiation and were required to use contraception throughout the study and for 4 months after enrollment.

*Results and Conclusion*s. Although full trial data have not yet been published, early reports indicate that AF demonstrated a good safety profile, with no serious adverse events reported and good overall tolerability among participants. Clinically, AF showed a clear benefit over placebo in achieving resolution of diarrhea by the designated endpoints, day 5 for giardiasis and day 7 for amebiasis, defined as fewer than three loose stools in 24 h. Additionally, parasitological outcomes, including stool antigen clearance and microscopic evaluation, supported the efficacy of the treatment. These findings suggested that AF may be an effective and safe alternative therapy for protozoan gastrointestinal infections, especially in contexts where resistance to traditional drugs, like metronidazole, is a growing concern. The trial results provide a strong basis for future Phase III studies aimed at confirming the drug’s clinical utility and broadening its role as a repurposed antiparasitic agent.

### 4.3. **NCT02961829**—Multi Interventional Study Exploring HIV-1 Residual Replication: A Step Towards HIV-1 Eradication and Sterilizing Cure https://clinicaltrials.gov/study/NCT02961829 (Accessed on 20 July 2025)

*Rationale*. Despite the remarkable success of antiretroviral therapy (ART) in suppressing viral replication and restoring immune function, a definitive cure for HIV remains elusive. This is primarily due to the persistence of latent viral reservoirs, mainly located in long-lived CD4^+^ memory T cells and certain tissue compartments. For this reason, a multifaceted approach is increasingly considered essential to achieve an HIV cure.

Therefore, a combination strategy that simultaneously targets different mechanisms of viral persistence appears to be the most promising approach.

*Objectives*. This pilot proof-of-concept study aimed to explore the use of a combination of drugs to eliminate residual plasma viremia and reduce HIV reservoirs in patients on long-term ART. These include (i) enhancing antiretroviral therapy with Maraviroc and/or Dolutegravir, aimed at blocking viral entry and replication [53]; (ii) stimulating the immune system with a personalized dendritic cell vaccine derived from autologous HIV; (iii) reactivating latent virus using a latency-reversing approach focused on Class III histone deacetylase (HDAC) inhibition, like Sirtuin-1 [54]; and (iv) selectively reducing long-lived HIV reservoir cells, mainly located in central and transitional memory CD4^+^ T cells, by using AF [45,55].

*Treatment*. Adults with chronic HIV infection and stable viral suppression under ART were selected and assigned to one of six groups, each receiving a different combination of drugs over 48 weeks. The six groups were: (i) the control group which continued with the standard ART regimen therapy without any additional interventions, serving as a baseline for comparison; (ii) the ART Intensification group, which received dolutegravir and maraviroc; (iii) the ART Intensification + Nicotinamide group which received the same ART intensification (dolutegravir + maraviroc), along with nicotinamide, Sirtuin HDAC inhibitor; (iv) the ART Intensification + AF group for which the intensified ART was combined with AF; (v) the ART Intensification + Therapeutic Dendritic Cell Vaccine group for which the intensified ART was supplemented with an autologous dendritic cell-based vaccine; (vi) the Multimodal Intervention Group which received the most comprehensive treatment, combining all components: intensified ART, nicotinamide, AF, and the dendritic cell vaccine.

*Eligibility*. The study enrolled 30 patients. Participants eligible for inclusion in the study were adults aged 18 years or older with a documented diagnosis of HIV-1 infection. To qualify, participants needed to be on a stable Highly Active AntiRetroviral Therapy (HAART) regimen for at least two years, with no changes in their ART during the 24 weeks immediately preceding the screening.

In addition, all candidates had to demonstrate sustained virologic suppression, defined as an HIV-1 viral load below 50 copies/mL, without any two consecutive measurements exceeding this threshold in the past two years.

*Results and Conclusions*. While a full “cure” was not achieved yet, the early results obtained suggest that combining ART intensification, immune stimulation, and latency reversal could be a promising pathway toward reducing the HIV reservoir. However, more comprehensive analysis is needed.

## 5. Conclusions and Future Perspectives

It is nowadays widely recognized that improvement of currently available clinical protocols for treating cancer or different diseases can extensively benefit from multiple approaches. Among them, the repurposing of approved drugs for indications different from the original ones represents a reliable and efficient strategy coupling the likely rapid translation in the clinic with an economically sustainable profile. In this frame, inorganic approved medicines are suitable because of their chemical versatility and ability to target multiple biological substrates. In fact, the well-known high reactivity of metal centers can be conveniently exploited for the tailored treatment of various pathologies [56,57].

In such context, in this review, we provided an updated overview of the clinical trials investigating the repurposing of AF in oncological and non-oncological settings [58].

In detail, a search conducted on ClinicalTrials.gov using the entry “auranofin” as “other terms” identified fifteen studies. Out of these, six studies were excluded for the following reasons: two had been withdrawn prior to patient enrollment (the reasons are unknown), one was listed with an unknown status, one had not yet begun recruiting at the time of our search, and two were unrelated to the primary scope of this review (AF is used as a reference drug in rheumatoid arthritis conditions). The remaining nine trials were herein analyzed and discussed. Among these, six trials repurposed AF in various cancer types in mono- (Epithelial Ovarian, Primary Peritoneal, or Fallopian Tube Cancer, Chronic Lymphocytic Leukemia, paclitaxel-induced pain syndrome) and in combination- (lung, ovarian cancer, and glioblastoma) therapy. The other three trials target non-oncological diseases, namely amoebiasis, giardiasis, and HIV.

**Figure 4:**
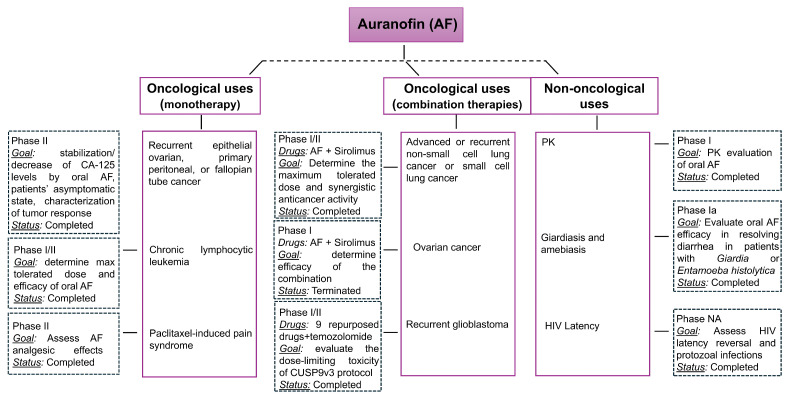
A flowchart of the clinical trial results of AF.

For the sake of clarity, a schematic flowchart of the clinical trial results is presented in Figure 4.

Three trials posted clinical results: NCT02063698, NCT 03456700, and NCT02736968.

The NCT02063698 focused on the potential of AF to counteract paclitaxel side effects, aiming to offer preliminary evidence for its use as a supportive care option in oncology. By comparing the outcomes between the AF and placebo groups, the study aimed to determine whether short-term use of AF could meaningfully prevent or reduce chemotherapy-induced pain. Although there is no conclusive evidence on the effectiveness of AF, the delay in the insurgence of pain observed in the treatment group provided proof that may warrant further investigation, perhaps with adjusted dosing regimens or extended treatment duration.

NCT03456700 is a Phase II study conducted by the Mayo Clinic evaluating the efficacy of AF in combination with sirolimus in patients with recurrent serous ovarian cancer. It was evidenced that, while the combination of AF and sirolimus was generally well tolerated, the modest clinical benefits observed do not support its adoption as a standard treatment for this patient population.

Trial NCT02736968 is a phase IIa study assessing the efficacy of AF in patients with either giardiasis or amebiasis, sponsored by the National Institute of Allergy and Infectious Diseases (NIAID). The obtained results indicate the potential of AF as a safe and effective treatment option for gastrointestinal protozoal infections, particularly in settings where resistance to standard therapies such as metronidazole is becoming more prevalent. The trial outcomes offer solid support for advancing to Phase III studies to further assess its clinical effectiveness and expand its application as a repurposed antiparasitic drug.

Although the available clinical trials on AF repurposing show encouraging safety and pharmacokinetic profiles, the efficacy outcomes are still being evaluated. In this context, limitations likely result from factors such as small patient cohorts, lack of biomarker-based selection, and suboptimal dosing regimens related to AF’s long half-life and complex pharmacokinetics [59]. The prolonged plasma half-life of AF, reported to range between approximately 15 and 35 days depending on dose and biological compartment [6,60,61], poses significant challenges for dose optimization. Such extended persistence may lead to drug accumulation, raising concerns about long-term tolerability. Conversely, for short-term applications, the long half-life may delay achievement of steady-state concentrations, limiting immediate pharmacodynamic effects. Future studies should therefore incorporate pharmacokinetic modeling and therapeutic drug monitoring to refine dosing strategies and ensure an optimal balance between efficacy and safety in AF-based treatments.

Similar considerations have been discussed in recent studies exploring predictive biomarkers and combinatorial strategies for AF, where the expression of thioredoxin reductases 1 (TXNRD1) and other redox-related targets was found to influence therapeutic response [62,63].

In line with the recommendations proposed by Kupersmith and Jette [64], future clinical trials investigating AF repurposing should aim for more robust and wider applications. Particular attention should be given to optimizing dose regimens based on translationally relevant preclinical models, expanding eligibility criteria to better reflect real-world patient diversity, and integrating long-term follow-up to capture delayed toxicities or secondary effects. Such improvements could strengthen the translational potential of AF across different therapeutic areas [64,65].

In general, the field of drug repurposing has produced several notable successes [66,67], such as sildenafil (initially developed for angina, later repurposed for erectile dysfunction), minoxidil (from antihypertensive drug to treatment of androgenetic alopecia), and raloxifene (developed for osteoporosis and subsequently used in breast cancer prevention). Nevertheless, repurposing does not always succeed, and it may be due to insufficient efficacy, unforeseen toxicities, or pharmacokinetic limitations. For example, topiramate, an originally developed antiepileptic drug, was repurposed for the treatment of inflammatory bowel disease (IBD); however, the results showing promising efficacy in a rodent model of IBD were not confirmed in a subsequent retrospective cohort study. These examples underscore both the promise and the inherent risk of this strategy.

In this context, the long-established safety record and well-characterized pharmacology of AF make it an appealing candidate for repurposing, but its clinical performance to date has been variable and needs to be further investigated. Consequently, while AF remains a compelling candidate worthy of continued investigation, its successful repurposing will require rigorous, adequately powered clinical trials, careful patient selection, and possibly rational drug combinations.

## Figures and Tables

**Table 1:** Clinical trials involving Auranofin (from https://clinicaltrials.gov/, accessed on 20 July 2025).

ClinicalTrials.gov Identifier	Indication	Sponsor	Status ^a^	Phase ^b^	Results ^c^
NCT01747798	Epithelial Ovarian, Primary Peritoneal, or Fallopian Tube Cancer	Mayo Clinic	C (2019)	Early 1 (0)	N/A
NCT01419691	Chronic Lymphocytic Leukemia (CLL)	University of Kansas Medical Center	C (2016)	2	N/A
NCT02063698	Paclitaxel-Induced Pain Syndrome	Mayo Clinic	C (2019)	2	Y
NCT01737502	Lung cancer	Mayo Clinic	C (2024)	1/2	N/A
NCT03456700	Ovarian Cancer	Mayo Clinic	T (2025)	2	Y
NCT02770378	Glioblastoma	University of Ulm	C (2021)	1/2	N/A
NCT02089048	PK	National Institute of Allergy and Infectious Diseases (NIAID)	C (2017)	1	N/A
NCT02736968	Amoebiasis or giardiasis	National Institute of Allergy and Infectious Diseases (NIAID)	C (2023)	2	Y
NCT02961829	HIV	Federal University of São Paulo	C (2020)	N/A	N/A

^a^ C = completed (The study has ended normally, and participants are no longer being examined or treated); T = terminated (The study has stopped early and will not start again. Participants are no longer being examined or treated). The year of the last update is reported in bracket in the “Status” column. ^b^ N/A = Not Applicable. ^c^ Y = Results published; N/A = Results not Available.

## Data Availability

No new data were created or analyzed in this study.
